# Investigation of Influencing Factors on the Measurement Signal of a CMOS Image Sensor for Measuring Field Emission Currents

**DOI:** 10.3390/s25051529

**Published:** 2025-02-28

**Authors:** Matthias Hausladen, Andreas Schels, Ali Asgharzade, Philipp Buchner, Mathias Bartl, Dominik Wohlfartsstätter, Simon Edler, Michael Bachmann, Rupert Schreiner

**Affiliations:** 1 Faculty of Applied Natural and Cultural Sciences, Ostbayerische Technische Hochschule Regensburg, 93053 Regensburg, Germany; ali.asgharzade@oth-regensburg.de (A.A.); philipp.buchner@oth-regensburg.de (P.B.); mathias.bartl@oth-regensburg.de (M.B.); rupert.schreiner@oth-regensburg.de (R.S.); 2 Ketek GmbH, 81737 Munich, Germany; andreas.schels@ketek.net (A.S.); dominik.wohlfartsstaetter@ketek.net (D.W.); simon.edler@ketek.net (S.E.); michael.bachmann@ketek.net (M.B.)

**Keywords:** field emission imaging, field emission, CMOS sensor, electron detector

## Abstract

We use optical CMOS image sensors for spatially and time-resolved measurement of the emission currents of field emission cathodes. The measured signal depends, on the one hand, on the emission current that flows from the cathode surface through the vacuum to the sensor surface. On the other hand, it is influenced by other variables, such as the extraction voltage, which accelerates the electrons towards the sensor surface, and the exposure time set on the sensor. In this article, these influencing factors on the measured pixel signals of a CMOS image sensor are examined in detail. In the first step, an equation is formulated that describes the signal measured by the sensor as a function of the emission current from a field emission tip, with the acceleration voltage and the exposure time as parameters. In the next step, we explain how the sensor signal is determined from the captured images. We then conduct experiments with a segmented field emission array consisting of 2 × 2 individually addressable emitters, where the voltage and currents for each emitter are known. The sensor signals are then measured for various voltages and currents and compared with the theoretical predictions. Thus, we demonstrate that, for a known voltage, the sensor signals obtained from the images can be corrected using the theoretical correlation, allowing the sensor signal to be used to measure the emitter current. This method can also be applied to investigate field emission arrays with many tips, provided that the emission spots on the CMOS sensor images can be clearly distinguished.

## 1. Introduction

A suitable measurement method is essential for determining the emission current distribution of field emission arrays (FEAs), both spatially and temporally. Our research group has started to develop a system using a Raspberry Pi High Quality Camera [1] which is equipped with a Sony IMX477 image sensor [2]. This system can be used for the analysis of silicon FEAs [3,4]. It does not require additional electron–photon converters and provides a resolution comparable to the smallest grain sizes of commercially available phosphor screens [5,6,7,8]. Due to the possibility of adjusting the exposure time of the CMOS sensor, the dynamic measuring range is variable. By combining different exposure times, both low- and high-emission currents can be measured. Recently, we improved the measurement method by metal coating of the image sensor [9]. This modification increased the resistance to permanent pixel damage and eliminated the side effect of locally charged microlenses. However, a non-linear influence of the field emitter voltage both for the uncoated, as well as for the coated sensor, was experimentally observed. In the following, we will examine the dependence of the sensor signal on the influencing factors of voltage, current, and exposure time. Our aim is to understand the relationship between the sensor signal and the emission current when voltage and exposure time are varied. This understanding is very important for the practical application of this method. In our previous investigations, the prerequisite for the practical applicability of the method was that the voltage difference between all individual emitters of an array is approximately the same.

For this purpose, we first look at the theoretical mathematical relationship between the sensor signal and the emission current, the applied voltage, and the set exposure time. Here, we also describe in detail how the sensor signal is obtained from the image data. We then carry out experiments with different voltages, currents, and exposure times using a segmented cathode with four individually controllable emitter tips.

## 2. Theoretical Description of Signal Generation in the Sensor as a Function of the Emission Current

In the following, the mathematical description of the formation of the sensor signal is derived and explained. For more clarity on the absorption relation in the derivation, we point out that in Chapter 3.1 provides a focused ion beam cross-section of the Sony IMX477 image sensor [2]. Subsequently, we describe how the method mathematically utilizes the measured sensor signals to determine the optically derived individual currents from the acquired images when applied to FEAs with numerous emission sites [3,4,9].

### 2.1. Description of the X-Ray Signal Generated by the Incident Electrons

When moving free electrons are decelerated and deflected by and near atomic nuclei, they convert a part of their kinetic energy into photons. This phenomenon is called Bremsstrahlung and generates a continuous X-ray spectrum of radiation [10,11]. Assuming a single electron that converts its entire energy into a single photon determines the shortest possible wavelength *λ_min_* which can occur in the accruing X-ray continuum. Its value is calculated by the Duane–Hunt formula [11,12]:

(1) $$\lambda_{min}(U)=\frac{h\cdot c}{e\cdot U}$$

where *h* is Planck’s constant; *c* is the speed of light; *e* is the elementary charge; *U* is the acceleration voltage of the electron.

**Figure 1:**
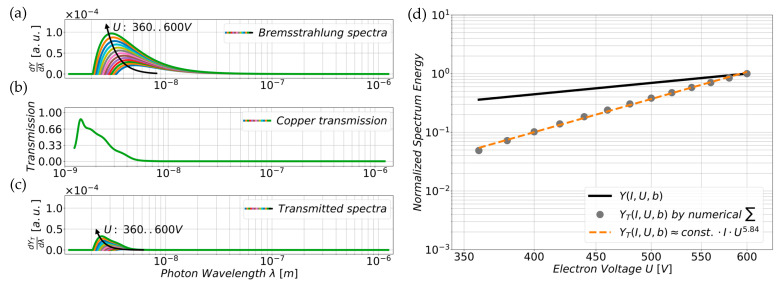
(**a**) Numerically computed intensity distributions (Equation (2)) of the generated Bremsstrahlung spectra *dY/dλ* for an exponent *b* = 2 and a constant current *I* = 1 µA within the voltage range of the experiment (*U* = 360–600 V, 20 V spacing ascending in arrow-direction). (**b**) Depicts the transmission curve for a Cu layer (*d* = 150 nm), calculated from the spline-interpolation of the discrete set of absorption coefficients *µ*(*λ*) of Hagemann [15,16], and considering the electron penetration depth *R*(*U*). The transmission curves were pointwise multiplied with the separate Bremsstrahlung spectra to obtain (**c**) the transmitted spectra *dY_T_*/*dλ* (Equation (5)), which penetrate to the image sensor pixels. (**d**) Shows the normalized total intensities of the numerically computed Bremsstrahlung spectra *Y*(*U*) and of the transmitted spectra *Y_T_*(*U*) as well as an approximation of the transmission spectra intensity (*Y_T_*(*U*) ≈ *const. ∙ I ∙ U*^5.84^) on double logarithmic axes.

According to Müller-Sievers [13], the intensity distribution of a continuous Bremsstrahlung spectrum can be calculated by Kramer’s law:

(2) $$\frac{dY\left(I,U,\lambda ,b\right)}{d\lambda }=C\cdot I\cdot \left(\frac{1}{\lambda_{min}\left(U\right)}-\frac{1}{\lambda }\right)\frac{1}{\lambda^{b}}$$

with *dY/dλ* as differential photon intensity per wavelength interval. *C* combines several natural constants and constant parameters like Kramer’s constant, the atomic number of the target material, etc. [13]. The emission current is expressed by *I* while *λ* represents the momentary photon wavelength under consideration within an X-ray continuum. The exponent *b* has in general a value of 2 for Kramer’s law but varies between 1.8 and 4.0 for soft X-rays as experiments have shown [13]. Later, we reference *b* as the voltage exponent due to the following equation transformations. Figure 1a shows graphical representations of 13 numerically computed Bremsstrahlung continua (Equation (2)), which were calculated for a variety of voltages between *U* = 360 and 600 V (20 V spacing). This is because this voltage range was applied to our field emission tips during the experiments (ref. 5.2). Assuming the exponent *b* as a constant parameter and since the emission current *I* affects the intensity but not the spectral distribution, which is defined by the acceleration voltage *U*, their values were defined as *b* = 2 and *I* = 1 µA. An expression for the total intensity *Y* of a Bremsstrahlung continuum of a certain voltage *U* is obtained by integrating Equation (2) within defined limits. This yields the following term, which is dependent on the emission current *I*, the extraction voltage *U*, and the exponent *b*:

(3) $$Y\left(I,U,b\right)=\int_{\lambda_{min}}^{\infty }\frac{dY\left(I,U,\lambda ,b\right)}{d\lambda }d\lambda =\frac{C}{b\left(b-1\right)}\cdot \frac{I}{\lambda_{min}^{b}\left(U\right)}$$

Inserting Equation (1) for *λ_min_*, the analytical formula transforms as follows:

(4) $$Y\left(I,U,b\right)=const.\cdot I\cdot U^{b}$$

The Bremsstrahlung must also pass through the target material where the X-rays are generated before reaching the image sensor pixels. Consequently, the wavelength-dependent absorption of the target material needs to be considered by multiplying the Bremsstrahlung spectrum of Equation (2) with the absorption relation [14]. The factorial transmission curve for a Cu coating with a thickness of *d* = 150 nm was calculated from the spline-interpolation of the discrete absorption coefficients *µ*(*λ*) of Hagemann [15,16], assuming a constant initial intensity of *dY*/*dλ* = 1 W/m^2^. Note that the transmission curve also takes into account the electron penetration depth *R(U)* by including the Kanaya–Okayama model [17,18] in the equation. Finally, we obtain an expression for the wavelength-dependent transmitted Bremsstrahlung spectrum *dY_T_*/*dλ*:

(5) $$\frac{dY_{T}}{d\lambda }\left(I,U,\lambda ,b\right)=\frac{dY\left(I,U,\lambda ,b\right)}{d\lambda }\cdot e^{-µ\left(\lambda \right)\cdot (d-R\left(U\right))}$$

Figure 1c depicts the transmitted Bremsstrahlung spectra obtained from Equation (5), using the same voltage range and parameters as for Equation (2) above. Note that the absorption effect of the microlenses is not considered as the exact material is unknown. The Bayer-Filters are also neglected as the filter-characteristics in the datasheet are only given for the visible EM-spectrum of *λ* = 400–700 nm [2,19].

For later comparison with the experimental data, the normalized total intensities of the generated Bremsstrahlung continuum were numerically computed using Equation (4) and are plotted in Figure 1d using *b* = 2 and *I* = 1 µA (*Y*(*I*, *U*, *b*), solid black line). Since the wavelength-dependent absorption coefficients *µ*(*λ*) of Cu [15,16] are only available as a discrete set of sampling points, and not as an analytical function, Equation (5) is no longer applicable for the analytical integration of the filtered spectra. Therefore, the filtered spectra were numerically integrated as a sum over the wavelengths ranging from *λ_min_* ≈ 1.24 nm to *λ_max_* ≈ 1.24 µm. The boundaries correspond to an electron energy interval from *E_min_* = 1 keV to *E_max_* = 1 eV, encompassing the relevant range of the X-ray continuum to numerically integrate its total intensity *Y_T_*(*I*, *U*, *b*) (Figure 1d, gray dots). It is not obvious that Equation (4) is also valid here due to the influence of the absorption. However, when fitting the total intensities *Y_T_* with Equation (4) for the voltage range *U* between 320 V and 600 V, we obtain a good approximation of *Y_T_*(*I*, *U*, *b*) (Figure 1d, orange dashed line) for *b* = *b_Fit_* = 5.84 with respect to the numerical integration:

(6) $$Y_{T}\left(I,U,b\right)\approx \;const.\cdot I\cdot U^{b}$$

### 2.2. Generation of the Electrical Sensor Signal for One Electron Emission Spot

Electrons that hit the image sensor at a certain spot generate an X-ray signal, which in turn generates a signal *S_Raw_*_0_ at this spot on the captured image. A spot usually comprises several pixels, which are then added together. In [4], we showed that there is a linear relationship between the set exposure time *t_E_* and the measured sensor signal:

(7) $$S_{Raw0}\left(I,U,b,t_{E}\right)\propto I\cdot U^{b}\cdot t_{E}$$

The raw spot sensor signal *S_Raw_*_0_ is directly proportional to the current *I* and the exposure time *t_E_*. The electron voltage *U*, on the other hand, influences *S_Raw_*_0_ based on a power law with the voltage exponent *b*. Typically, the *S_Raw_*_0_ value of the longest exposure time *t_E_*_,*Ref*_ is used because it provides the highest signal-to-noise ratio. However, the spot signal *S_Raw0_* can be overexposed. In this case, the *S_Raw_*_0_ value of a shorter, not overexposed exposure time *t_E_*_,*nOE*_ is taken and scaled up using the proportional upscaling factor *f_u_*(*t_E_*_,*nOE*_) *= t_E_*_,*Ref*_/*t_E_*_,*nOE*_:

(8) $$S_{Raw}\left(I,U,b,t_{E,nOE}\right)=S_{Raw0}\left(I,U,b,t_{E,nOE}\right)\cdot f_{u}(t_{E,nOE})$$

By inserting Equation (7) into Equation (8) and dividing by the voltage (*U^b^*), we obtain the following expression for the voltage-independent spot signal *S*:

(9) $$S\left(I\right)=\frac{S_{Raw}\left(I,U,b,t_{E,nOE}\right)}{U^{b}}\propto I\cdot t_{E,Ref}$$

Note that *t_E_*_,*Ref*_ is a global constant due to the upscaling factor *f_u_*, eliminating the exposure time dependence of sensor signal values.

### 2.3. Determination of Individual Currents for Field Emission Arrays with Multiple Emission Spots

If multiple spots appear in an image when measuring a field emission array, the procedure described above is carried out for each individual emission spot. In order to be able to distinguish the individual spots, they are numbered consecutively and Equation (9) is indexed, yielding the following expression:

(10) $$S_{i}\left(I_{i}\right)=\frac{S_{Raw,i}\left(I_{i},U_{i},b,t_{E,nOE,i}\right)}{U_{i}^{b}\cdot t_{E,Ref}}\propto I_{i}$$

From the individual spot signals *S_i_* of the emitter tips, normalized share factors are determined for each spot (*F_i_*). This is achieved by dividing their individual spot sensor signal *S_i_* by the total sensor signal of all spots on an image. This yields a relation for the share factors *F_i_*, which are only dependent on the electron spot current *I_i_*:

(11) $$F_{i}(I_{i})=\frac{S_{i}(I_{i})}{\sum_{i=1}^{n}S_{i}(I_{i})}$$

By multiplying the computed share factors *F_i_* with the electrically measured total current (*I_Total_*), the individual optically mapped (OMap) tip currents *I_OMap i_* are obtained:

(12) $$I_{OMapi}(I_{i})=I_{Total}\cdot F_{i}(I_{i})$$

The total current *I_Total_* flowing into the field emission cathode can be easily determined by measurement. By using Equation (12), it is now possible to determine the distribution of the total current to the individual emitters of an array.

## 3. Experimental Setup for Carrying out the Experiments to Measure Field Emission Currents with the CMOS Sensor

### 3.1. Overview over the Experimental Setup

**Figure 2:**
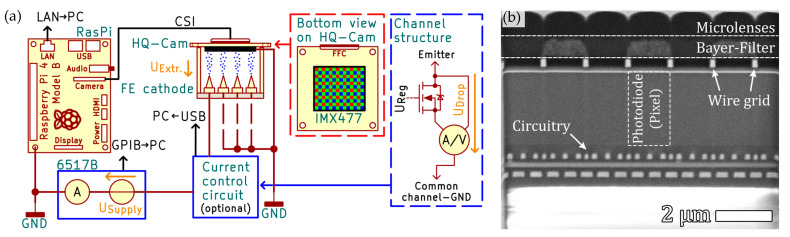
(**a**) Connection schema of the generic experimental setup. The individual instruments are controlled by a PC via different busses (Ethernet/LAN, GPIB, USB), represented by the black lines. The electrical single wire connections are depicted by the brown lines. The FE cathode is mechanically clamped to the image sensor (IMX477) surface with a tip-to-surface distance of ≈ 60 µm. The red dashed frame illustrates the Bayer-Filter of the image sensor. The semi-transparent current control circuit is used for validating the linearity of the exposure time (*t_E_*) influence but removed for measurements observing the influence of the emission current (*I*) and the extraction voltage (*U*). Its internal structure is shown by the semi-transparent blue dashed frame. The 6517B serves as high-voltage supply for the field emitter under test and measures its total emission current (*I_Total_*). (**b**) FIB cross-section of the layer stackup of a Sony IMX477 CMOS image sensor of a commercially available HQ-Cam. The microlenses and the Bayer-Filter have a thickness of ≈1 µm. The photodiodes underneath possess a thickness of ≈ 2.5 µm.

The experimental setup consists of a specially prepared silicon field emission cathode. This cathode offers the option of setting different currents and voltages on 4 individual addressable emitters. This means that four different emission spots can be generated on the CMOS sensor independently of each other. The cathode is mechanically fixed to the image sensor of the 150 nm Cu-coated Raspberry Pi High Quality Camera [1] (HQ-Cam, Figure 2a). The Cu coating improves the protection of the image sensor against physical pixel damage and prevents lens charging of the sensor surface under electron bombardment due to a grounded metallic layer. To give an impression of the relationship between the 150 nm thick Cu coating and the internal sensor dimensions, Figure 2b illustrates a cross-sectional view after applying focused ion beam (FIB) milling. Moreover, it provides a detailed insight into the internal structure of the image sensor. A detailed description of the HQ-Cam disassembly and modification process can be found in [9]. This stack consisting of a field emission cathode and image sensor is then installed in a vacuum chamber with a pressure of approximately ≈ 10^−7^ mbar. The HQ-Cam is connected to the Raspberry Pi 4 (RasPi) via the camera serial interface (CSI) using flange feedthroughs. The individual emitters are each individually connected to a Keithley 6517B, which measures the electrical total current of the cathode while its integrated high-voltage source serves as high-voltage supply. This means that both the current and the extraction voltage of each emitter are known individually. An optional multi-channel current control circuit [20] can be inserted between an emitter tip and the voltage supply to maintain a constant field emission current per tip. Each channel measures the momentary current value, which is used to determine the necessary gate voltage *U_Reg_* for the regulation MOSFET (Figure 2a, Channel Structure). Simultaneously, each channel tracks the voltage drop across the regulation MOSFET. By this, the effective extraction voltage can be calculated by *U_Extr._ = U_Supply_–U_Drop_*. This was used to validate the linear influence of the exposure time *t_E_* (ref. 5.1.2) and was removed in the measurements regarding the influence of emission current *I* and extraction voltage *U* (ref. 5.2). The separate measurement and source devices are coordinated and managed by a PC, executing our measurement software [21], utilizing different buses for communication (Ethernet/LAN, GPIB, USB).

### 3.2. Acquisition Hardware for the CMOS Image Sensor

We use a Raspberry Pi 4 [22] (RasPi) microcomputer as acquisition hardware for our setup. To communicate with the RasPi, its Ethernet port is connected to the same local area network (LAN) as the measurement PC (Figure 2a). The RasPi provides a hardware-accelerated encoder for JPEGs, which makes capturing 8-bit JPEGs fast but not applicable due to the lossy compression algorithm. Furthermore, gamma-correction and other forms of image processing are applied automatically, resulting in a non-linear sensor signal response. To overcome this issue, camera tuning files [23,24,25], one of which is optimized for linear signal response, can be loaded during camera initialization. By this, the HQ-Cam is configured to return Bayer-encoded RAW images with 12-bit values per pixel and linear signal response with respect to the exposure time. For automated image capturing by our measurement software [21], a Python server-script (PyCam2-Server V1.0.0.0; executed by the RasPi) was developed [19]. This server-script manages the HQ-Cam and the network communication with the PC. To capture an image sequence of a single datapoint, the PC sends an instruction to the RasPi, which allows specification of the exposure times *t_E_* to be captured as well as the number of images per exposure time. Note that the returned images are encoded Bayer-data [26,27] which need to be decoded during post-processing. After capturing an image sequence, the Bayer-data are firstly archived on the RasPi and subsequently downloaded by the PC. Images captured in full resolution of the sensor require significant disk space and considerable time for processing. Therefore, the server-script is able to crop the encoded RAW Bayer-data [26,27] into smaller images. Depending on the image region of interest, the image size can be reduced significantly, resulting in a corresponding reduction in disk space and computing resources required. For example, the images analyzed in this article have a resolution of 1200 × 1200 px^2^, reducing the file size by a factor of ≈ 8.5 compared to a full resolution frame (4056 × 3040 px^2^, RAW image file size ≈ 35 MB). In order to expedite the image saving process further, the PyCam2-server is able to create a random-access-memory-disk (RAM-disk) of selectable size. In addition to the speed aspect, the lifespan of the SD card is extended by minimizing the number of write-accesses to the SD card’s memory cells.

### 3.3. Field Emission Cathode for Generating Several Individually Controllable Emission Spots on the CMOS Sensor

**Figure 3:**
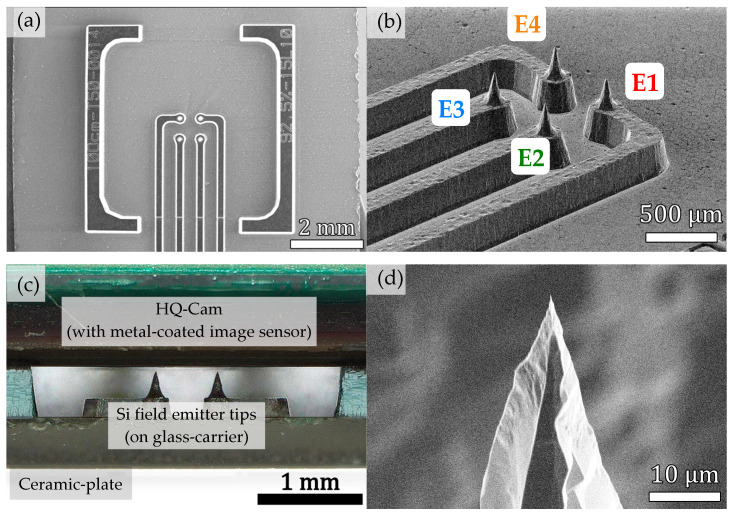
SEM images from (**a**) top view and (**b**) oblique view (with labels) on the segmented cathode with individually addressable emitter tips. (**c**) Side view of the cathode, which is clamped onto the image sensor surface using a ceramic-plate. The distance between tips and metal-coated image sensor surface is ≈60 µm. (**d**) Closeup SEM image of a sharpened field emitter after wet-chemical post-treatment.

For our experiments on field emission currents, a segmented field emission (FE) cathode with 4 individual addressable emitter tips in a 2 × 2 arrangement was fabricated (Figure 3a,b) [28]. The field emitters are surrounded by bracket look-a-like structures which define the distance between tip and metal-coated image sensor surface, which has a value of ≈ 60 µm (Figure 3c). Due to the ability to individually address each field emitter, we can measure the current and the extraction voltage of each single tip separately. The substrate of the FE cathode is n-doped silicon (phosphorus 5–10 Ωcm, orientation <100>, substrate thickness 525 µm), which is anodically bonded to a borosilicate glass-carrier (BF33, thickness 500 µm). The material stack was then processed by laser-micromachining, producing the fundamental cathode structure. However, melting effects of the laser process yield a rough surface and blunt field emitter cones. Therefore, wet-chemical post-treatment was carried out to turn the blunt cones into sharp field emitter tips. First, SiO2 is removed by an HF dip. A subsequent TMAH (25 %, 85 °C, 15 min) etch-step then transforms the blunt cones into sharp field emitting tips (Figure 3d). A detailed description of the process can be found in [28,29].

## 4. Measurement Data Acquisition and Image Post-Processing

### 4.1. Measurement Data Acquisition

**Figure 4:**
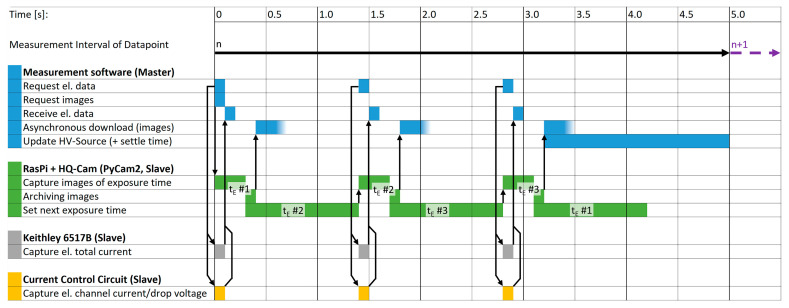
Sequence diagram of the software and instrument actions for one datapoint (*n*), capturing images at 3 different exposure times (*t_E_*), during a measurement cycle. Note that the exposure times *t_E_ #*1 … #3 are arbitrarily definable by the user. The download of the images is performed asynchronously in the background and can take longer than pointed out in the diagram, indicated by the fading out color gradient.

Our measurements consist of a defined number of datapoints which are acquired. For example, 50 datapoints are obtained if a single datapoint is acquired every 6 s for 5 min. Thereby, each datapoint follows the iterative operational sequence which is depicted in Figure 4. However, preliminary steps are executed prior to the first datapoint iteration. For this, the measurement software [21] instructs the PyCam2-server [19] to capture an image sequence of dark frames for each exposure time before any sources are turned on. This enables the detection of a damaged image sensor if their mean blacklevel is unusually high. This also allows for the dynamic elimination of the blacklevel value per exposure time from the images of each datapoint, which are utilized to obtain the spot signals during the image post-processing. Then, constant device-parameters (e.g., measurement ranges, voltage ranges, etc.) are sent to the devices and the iterative measurement commences. In order to have enough time for image acquisition, archiving, and downloading, the cycle-interval time for one datapoint needs to be set longer than the total time all actions consume. Figure 4 illustrates the chronological cycle-sequence of the instrument actions for a single datapoint *n*, in which 3 images each at 3 different exposure times (*t_E_*) are captured. By requesting the electrical data and images, a datapoint-sequence starts. Due to a maximum framerate of 10 frames per second, each image requires 100 ms of acquisition time. Exposure time (*t_E_*) changes take ≈ 1 s per change, which is defined by the open-source camera library Libcamera [23] used by our server-script. To minimize the time consumed for *t_E_* changes, the first *t_E_* of the subsequent datapoint *n + 1* is preset after the last image of datapoint *n* has been captured. Changes in *t_E_* enhance the dynamic range, or the measurable current range. This is often necessary due to the typically inhomogeneous current distribution among the individual emission sites of an FEA [3,4,9]. This allows for the identification of the image with the highest signal-to-noise ratio (SNR) and without overexposure for the sensor signal of the emission current of an emitter tip. Note that, as a rule, multiple images per *t_E_* are captured—we typically configure 3 images—to reduce fluctuations by averaging them into one image during post-processing.

Subsequently, the individual image archives of the different *t_E_* are downloaded asynchronously while the source devices are instructed to apply the supply value of the next datapoint *n* + 1. Finally, the measurement software awaits the end of the datapoint cycle-interval, acting as settle time for changed supply values. The electrical data, on the other hand, are measured and returned quickly in comparison to the images. For a high time–domain correlation between image and electrical data, separate electrical datapoints are measured for each *t_E_*. After a measurement procedure, a collection of RAW image archives and data files, containing the electrically measured data, is obtained.

### 4.2. Image Post-Processing to Obtain the Field Emission Spot Signals from the Images

The goal of image post-processing and analysis is to determine the spot parameters, particularly their image position (xy-coordinates) and their raw spot signal. For this purpose, the images are first converted into smaller portable network graphics (PNGs, lossless compression) and then analyzed by a separate algorithm. The obtained information is then saved as files to enable its subsequent combination and calculation with the electrical measurement data. In the following, both steps, image conversion and data analyzation, are described in detail.

#### 4.2.1. Conversion of the RAW Bayer-Data to 16-Bit Grayscale PNG

To obtain viewable portable network graphics (PNGs), the encoded RAW Bayer-data undergo a step known as demosaicking [27]. In this process, each 3 consecutive encoded Bayer-bytes (24 bits) are reconverted into 2 × 12-bit grayscale values or 2 pixels. Their values exhibit an offset which is known as blacklevel, which is caused by the dark current of their photodiode [30]. The individual frames of an exposure time of a datapoint are then averaged. Before saving an averaged image as 16-bit grayscale PNG, their 12-bit pixel values are shifted into the 16-bit range. This is for display purposes only and is not necessary for the data analysis. The conversion to PNG format is performed to further reduce the file size of an image and ensures that the images can be opened with a standard image viewer. The shift of the pixel values, however, is necessary because 12-bit values always appear black in a 16-bit grayscale PNG. Therefore, the value shift enables the identification of the region of interest, which is configured fixed, from an initial measurement with full-resolution images.

#### 4.2.2. Obtaining the Emission Spot Signals from the Image Data

**Figure 5:**
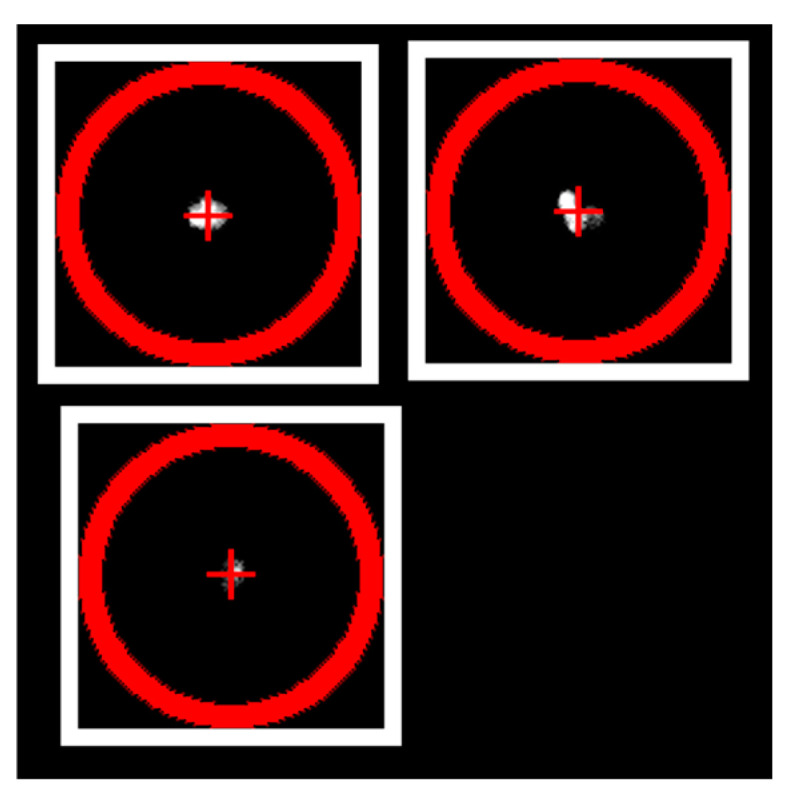
Exemplary region of interest of a 16-bit grayscale PNG captured by the image sensor, depicting 3 FE spots. The red crosses indicate the group-coordinates (x, y) and the red circles their assignment tolerance radii. The white frames enclose the rectangular area whose sum of pixel grayscale values results in a spot sensor signal. Note that circle diameter and rectangle side length actually have the same value but were drawn with different diameter/side length for display reasons.

The image data analysis script [19] imports all averaged 16-bit PNGs, sorted by their exposure time *t_E_* and in the order of the temporal sequence of capturing. The images with the highest sensor signals correspond to the longest *t_E_* and are most suitable for generating threshold images (*t_E_*_,*Ref*_). These images are forwarded to a spot detection algorithm which yields a set of center-coordinates and radii of the minimum enclosing circles of all detected spots in each frame. The script iterates then through all identified center-coordinates on each single image and assigns them to a group-coordinate (x, y). A group-coordinate, represented by the red crosses in Figure 5, is maintained across all images in the temporal sequence. The assignment itself is accomplished by checking if a spot’s center-coordinate in an image is within a parameterized tolerance radius (Figure 5, red circle) to an already known group-coordinate. If this applies, that center-coordinate is assigned to the corresponding group-coordinate; otherwise, a new group-coordinate is created. Once all spots have been aggregated, the group-coordinates are sorted from top left to bottom right. This enables easier allocation between the spot signals and their corresponding measured emission current. Then, the raw sensor signals of all spots are determined for each datapoint. A raw spot signal results by summarizing all pixel grayscale values within a parameterizable and rectangular area around the group-coordinate (Figure 5, white frames). The size of the area does not affect the sum of the raw spot signal, provided that it fully encloses the emission spot in the image and does not overlap with other spot areas. When calculating the integral spot signal, the individual pixel values are checked for overexposure by whether their grayscale values are too close to the 16-bit maximum value. The raw spot sensor signal is added to a separate data array if it is not overexposed. If the signal shows overexposure but remains within a configurable tolerance, it is also included in this array. This separate data array is designated to hold only non-overexposed or acceptably overexposed spot sensor signals. In case of unacceptable overexposure of a spot, the next shorter exposure time is checked recursively by the same procedure until an exposure time *t_E_* without overexposure is found (*t_E_*_,*nOE*_). The corresponding raw spot signal value is then linearly interpolated by multiplying with the upscaling factor *f_u_* = *t_E_*_,*Ref*_/*t_E_* and attached to the non-overexposed data array. Finally, as an intermediate step, the analyzed image data are saved as files on a hard drive, organized according to their respective group-coordinates. These files are then imported along with the electrical measurement data by user-defined data evaluation scripts, which, for instance, compute the optically mapped currents and then visualize the data. An example of a data evaluation script (V1.0.0.0) can be found in the GitHub repository [19].

## 5. Experiments and Results

To experimentally investigate the relationship between the spot signal determined at the sensor and the operating parameters set at the field emitter, a series of experiments were carried out. Initially, the influence of constant visible light irradiation on the exposure time was investigated by loading both the default tuning file and the linear tuning file during the initialization of an uncoated image sensor. This experiment was then repeated with a 150 nm Cu-coated image sensor, irradiating the sensor with a constant field emission electron current. Note that the Cu-coated image sensor was always initialized with the linear tuning file. A follow-up experiment, also using the Cu-coated image sensor, was conducted with a fixed exposure time *t_E_*. In this setup, various extraction voltages *U* were applied to emit different FE currents *I*. This approach allows for the investigation of their influence on the raw spot signal *S_Raw_*_0_.

### 5.1. Influence of the Exposure Time on the Sensor Signal

#### 5.1.1. Investigation of the Dependence of the Sensor Signal on the Exposure Time When Irradiated with Visible Light

**Figure 6:**
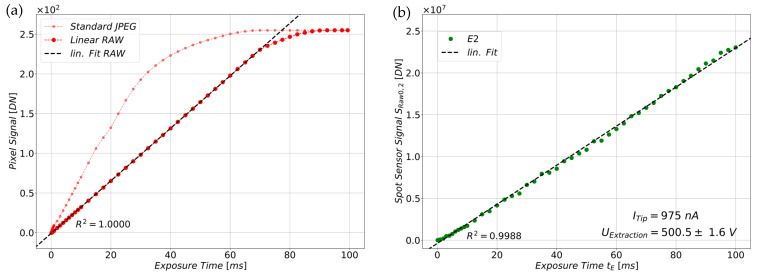
(**a**) Mean grayscale value of a pixel using the default tuning file (Standard JPEG) and the linear tuning file (Linear RAW) under cold-white light illumination (LED-strip, *P_el._* = 1.5 W). The signal of the JPEGs is quasi-linear but curved due to non-linear post-processing (e.g., gamma-correction). The RAW images are linear over the exposure time *t_E_* and saturate in 4 different regimes due to the influence of the Bayer-Filter (red, green, blue). (**b**) Depicts the linear influence of the exposure time *t_E_* on the spot sensor signal when tip E2 emits a constant current of 975 nA on the Cu-coated image sensor surface. Note the low fluctuation of the extraction voltage, which is why *U_Extraction_* is assumed as quasi-constant.

The following section investigates whether the linear tuning file produces a linear signal response compared to the standard sensor configuration (default tuning file) under light irradiation. For this purpose, an uncoated HQ-Cam was encased by an opaque housing, isolating the image sensor from ambient light. An LED-strip (cold-white, const. *P_el._* = 1.5 W) inside the housing was used as the designated light source while capturing datapoints between *t_E_* = 0.1 ms and *t_E_* = 100 ms. These measurements were carried out for both the default tuning file, yielding standard JPEGs, and the linear tuning file, yielding linear RAW images. For comparative purposes, the standard JPEGs (8-bit) were converted from the RGB color space (red, green, blue) into grayscale images to match the color space. The linear RAW images were already grayscale images but were converted from 12-bit to 8-bit to match the value range. Figure 6a illustrates the average pixel values of the images as digital numbers (DNs) over their exposure time *t_E_*. It can be seen that the RAW images are clearly linear up to a *t_E_* of ca. 70 ms and bend then into saturation in four linear regimes. The stepwise saturation is a consequence of the Bayer-Filter [2,27] (Figure 2a, Bottom View HQ-Cam), which generates distinct filter-spectra for each color filter (red, green, blue) under visible light. This produces disparate signals in the corresponding pixels behind the differently colored filter tiles. This is normally corrected by auto-white-balancing (AWB), which is deactivated by initializing the sensor with the linear tuning file. The offset of the linearized data was removed by subtracting the sensor’s mean blacklevel, which was determined from a set of dark frames captured at *t_E_* = 0.1 ms in complete darkness (light source turned off). To quantify how well the assumption of a directly proportional influence of the exposure time *t_E_* applies to the sensor signal, we calculated the coefficient of determination for the linear fit between 0.1 ms and 70 ms, yielding a value of *R^2^* = 1.00. The JPEG sensor signal, on the other hand, possesses a quasi-linear shape in the beginning, but starts bending at around 30 ms, reflecting all the non-linear post-processing steps like gamma-correction [31]. The graph also shows a small shift at around 20 ms, which is caused by the auto-gain-correction (AGC) algorithm. The AGC is enabled by default but is deactivated when initializing the image sensor with the linear tuning file.

#### 5.1.2. Investigation of the Dependence of the Sensor Signal on the Exposure Time Under Constant Electron Irradiation

The influence of the exposure time *t_E_* was re-examined, this time irradiating a Cu-coated image sensor with a constant field emission current. For this experiment, different constant emission currents (500 nA, 750 nA, 900 nA, 975 nA) were set on the current control circuit, which were emitted by the individual emitters of the segmented FE cathode. For overview reasons, only the spot sensor signal of tip E2 for a constant emission current of *I* = 975 nA is shown in Figure 6b. Given the low variance in the extraction voltage over the measurement duration, it can be reasonably assumed that the extraction voltage *U_Extraction_* is approximately constant too. Consequently, the sensor signal *S_Raw_*_0,2_ is only dependent on the exposure time *t_E_* as described by Equation (7). The linear relationship of *t_E_* is readily discernible and can be observed at all emitter tips, which is the case when their emission current and extraction voltage are high enough to generate a sufficient spot signal in the sensor. For quantification, the coefficient of determination for the influence of the exposure time *t_E_* under electron irradiation was calculated, yielding a value of *R*^2^ = 0.9988.

### 5.2. Influence of the Emission Current on the Sensor Signal at Different Extraction Voltages

**Figure 7:**
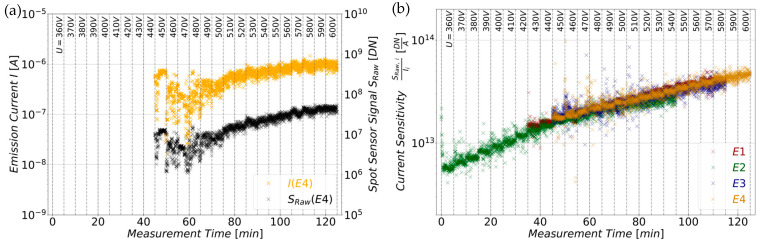
(**a**) Emission current *I*_4_ (colorized graph, left y-axis) and spot signal *S_Raw_*_,4_ (black graph, right y-axis) of emitter tip E4. Both show the same curve progression for voltage ranges with high fluctuations (e.g., 450–480 V) as well as in areas with low fluctuations (e.g., 580–600 V). This can be observed for all individual tips of the segmented cathode. (**b**) Dividing the sensor signals *S_Raw_*_, *i*_ by the corresponding emission currents *I_i_* of each tip yields their individual current sensitivities, which are approximately congruent for an emission at a specific voltage *U*. The graph, however, is not a constant but curved, indicating the non-linear influence of the extraction voltage *U*.

To measure the influence of the electron current and of the extraction voltage, the current control circuit was removed from the setup. This ensures that the current and the voltage are not externally influenced. Again, each tip was measured individually in sequence by applying a list of increasing voltages, ranging from 320 V to 750 V in 10 V steps while keeping the exposure time constant (*t_E_* = 100 ms). Each supply voltage was kept constant for 5 min while measuring 150 datapoints (0.5 Hz) for statistical reasons. Figure 7a shows the results of this measurements for emitter E4, excluding datapoints with raw sensor signals *S_Raw_*_0_ below the configured detection limit (insufficient) or with overexposure of more than 5%. The colorized graph depicts the FE current of emitter tip *I*_4_, which is related to the left y-axis. The black curve is the time-independent sensor signal *S_Raw_*_,4_, related to the right y-axis.

Both graphs are plotted against the measurement time, whereby the supply voltage *U* increases by 10 V each 5 min interval. These intervals are indicated by the vertical dashed gray lines. Both graphs clearly follow the same curve progression trends, which apply to regions with high fluctuations, (e.g., 450–480 V) as well as for regions with low fluctuations (e.g., 580–600 V). The same behavior was observed for the other three emitter tips too. However, it was observed that the resulting spot signals are strongly influenced by the voltage. For instance, E4 yields a sufficient and not overexposed spot signal *S_Raw0_*_,4_ in the voltage range of 450–600 V. In contrast, emitter E2 produces an adequate spot signal beginning at 360 V (sufficient) up to 540 V (overexposure). This circumstance is illustrated by Figure 7b, showing the current sensitivities of all four individual emitter tips. These were determined by dividing the individual time-independent spot sensor signals *S_Raw_*_, *i*_ by the corresponding electron emission currents *I_i_*. It is obvious that the current sensitivities of all tips are approximately congruent for a specific extraction voltage *U*. However, the sensitivities of the tips are not constant across the extraction voltage *U*. This allows us to infer how a sensor signal *S_Raw0_* behaves with respect to the extraction voltage *U* and whether it follows the voltage power law prediction given by Equation (7).

**Figure 8:**
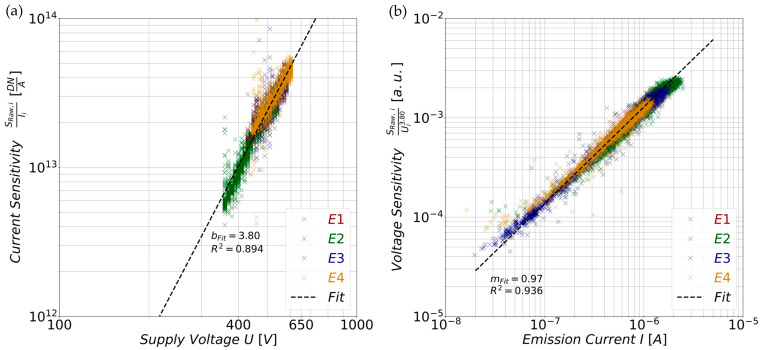
(**a**) Current sensitivities (*S_Raw, i_/I_i_*) of the segmented field emitters E1–E4 plotted against the extraction voltage *U* using double logarithmic scaling. A fit of the voltage exponent *b* of the extraction voltage *U* (Equation (7)) yields the optimum slope value *b_Fit_* = 3.80. (**b**) Using *b_Fit_* = *b* to calculate the voltage sensitivities *S_Raw, i_/U_i_^b^* reveals the proportional current dependency of Equation (7) on double logarithmic axes. A fit of these data yields an optimal slope value of *m_Fit_* = 0.97 with a coefficient of determination of *R^2^* = 0.94.

For this, the current sensitivities are plotted against their extraction voltage *U* while changing the axis scales to obtain a double logarithmic representation (Figure 8a). Because the current sensitivities show a few strong outliers, the dataset was filtered using the 50% interquartile interval with respect to each applied voltage. Subsequently, the filtered double logarithmic dataset was linearly fitted to obtain the optimum value of the exponent *b* (Equation (7)). The calculation yielded a value of *b_Fit_* = 3.80. Comparing Figure 8a with the numerical integrations of the transmitted spectra (Figure 1d, Equation (5)) reveals a very similar image. Both the measured data and the numerical integrated theoretical spectrum-energies exhibit slightly concave curve progressions. These are just barely discernable due to the linear fits determined for the numerical computations (Figure 1d, Equation (6)) and for the measurement data (Figure 8a, Equation (7)).

To quantize the approximation with respect to the voltage power law, we calculated the coefficient of determination, which has a value of *R*^2^ = 0.89. Consequently, the linear fits are not perfect but still a good approximation for a certain voltage range with respect to the current sensitivities. This issue arises from the fact that the filter curve of the target-material is only available as a discrete set of sampling points [15,16], and because of other unknown influences (Bayer-Filters and microlenses). These factors make an exact analytical integration of Equation (5) impossible, as mentioned earlier (ref. 2.1). This may also be an explanation for the discrepancy in the absolute fit values of the voltage exponents b of the theoretical prediction and experimental data. Therefore, the primary focus is on the qualitative curve progression, and further investigations are necessary in order to gain a deeper understanding of this phenomenon. Calculating the voltage sensitivity by dividing the individual *S_Raw, i_* by their extraction voltage using the power law *U_i_^b^* with *b* = *b_Fit_* = 3.80 reveals the influence of the electron emission current *I* (Figure 8b). The plot reveals the linear influence of the emission currents *I_i_* on the spot signal *S_Raw, i_* (Equation (7)), as the voltage sensitivities of the spots increase by a decade in value for each decade of current change. Note that Equation (7) describes the influence of the voltage with decreasing accuracy towards the edges of the voltage range. As a result, this also affects the voltage sensitivity in the same manner. Therefore, the voltage sensitivity was fitted within a current range from 1 ∙ 10^7^ A to 1 ∙ 10^6^ A, yielding a slope value of *m_Fit_* = 0.97. The computation of the coefficient of determination yields a value of *R^2^* = 0.94.

Finally, the current value of Equation (7) was back-calculated. For this purpose, a reference current value of *I_Ref_* = 975 nA was defined. Then, we identified the current value *I_Measured, i_* closest to *I_Ref_* from all measured current values *I_i_* of the individual emitter tips.

**Figure 9:**
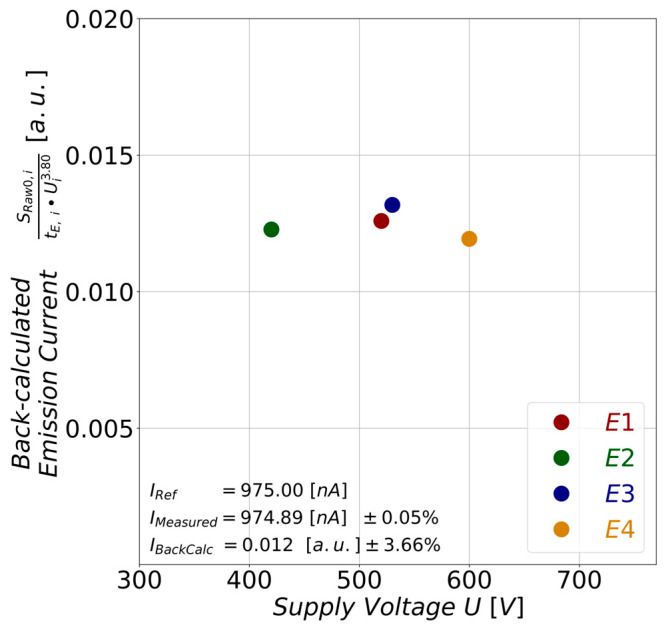
Back-calculated current values *I_BackCalc, i_* plotted against the supply voltages at which they were extracted. These currents were obtained from Equation (7) by inserting the following parameters: spot signal *S_Raw_*_0, *i*_, extraction voltage *U_i_*^3.80^, and exposure time *t_E_* = 100 ms. The parameter values were determined by identifying the measured emission current *I_Measured, i_* of the individual field emitters that are closest to a defined reference current of *I_Ref_* = 975 nA. The back-calculated current values do not match with reference current *I_Ref_* in absolute value but exhibit a low variation of ±3.66%. The mean values and standard deviations were determined for both the individual measured emission currents *I_Measured, i_* and the individual back-calculated currents *I_BackCalc, i_*. These are given in the diagram as *I_Measured_* and *I_BackCalc_*.

Subsequently, their corresponding values of the extraction voltage *U_Extr., i_*, the exposure time *t_E_*, and the raw spot signal *S_Raw0, i_* were inserted into Equation (7) to back-calculate *I_BackCalc, i_*. The resulting current values *I_BackCalc, i_* are plotted against their extraction voltage *U_Extr., i_* in Figure 9. The diagram illustrates that the original current can be derived from multiple sensor signals measured at a given current. Although the absolute values do not match, the low variation of approximately ± 3.5% indicates that the back-calculated currents are consistently close to one another. This offers the potential to calibrate the sensor, which enables direct current measurements via the image sensor.

## 6. Conclusions

In this article, an equation is formulated that describes the sensor signal as a function of the emission current from a field emission tip. To validate this equation, experimental measurement data were obtained from a segmented field emission cathode with four individually addressable emitters, whose emission currents and extraction voltages are known. The individual influencing factors—exposure time, emission current, and extraction voltage—were analyzed in detail. Their impact on the measured signal was examined, and the corresponding coefficients of determination were calculated.

In addition, we were able to back-calculate the current values for the individual emitter tips from the formulated equations, with the resulting values being close to each other and showing low variation. As a consequence, the sensor can be calibrated, which enables current measurements on field emitters to be performed directly via the image sensor in future.

Moreover, all necessary steps of our method, such as data acquisition and image post-processing, have been described in detail to support readers interested in replicating the measurement technique. To facilitate this, we have made all the self-developed source codes and hardware schematics publicly available on GitHub [19,20,21].

## 7. Outlook

Thus far, we have applied this method to silicon field emission arrays with rectangular tip arrangements with tip pitch distances of 67 µm [3], 250 µm [4], and 200 µm [9]. In the future, we also aim for the measurement of FEAs exhibiting other tip arrangements (hexagonal, parastichy, …) as well as with smaller tip pitches.

Moreover, we aim to further enhance our method [19] in the future. By choosing Python as our programming language, the individual software components can be easily transferred to different, more powerful hardware platforms. Additionally, the camera library Libcamera [23] provides the flexibility to manage various camera modules and image sensors. This simplifies both replacement of the acquisition hardware and replacement with different image sensors [32,33].

## Data Availability

The data contained in the images and plots in the article can be provided by the corresponding author. The source code of the measurement software (data acquisition) as well as the scripts for post-processing and evaluating the raw data are available via the referenced GitHub repositories or can also be provided by the corresponding author.
